# Paeoniflorin improves atherosclerosis by regulating the gut microbiota and fecal metabolites

**DOI:** 10.1128/msystems.00990-25

**Published:** 2025-08-15

**Authors:** Mengyao Xia, Zhiping Jiang, Bin Li, Yuna Liang, Hui Zhao, Dongmin Xie, Xianliang Song, Fei Ma

**Affiliations:** 1 Pharmacy Intravenous Admixture Services, The Affiliated Taian City Central Hospital of Qingdao University 230965, Taian, Shandong, China; 2 Oncology Surgery, The Affiliated Taian City Central Hospital of Qingdao University 230965, Taian, Shandong, China; 3 Ultrasound Diagnosis and Treatment Center, The Affiliated Taian City Central Hospital of Qingdao University 230965, Taian, Shandong, China; 4 Third level Hospital Assessment and Evaluation Office, The Affiliated Taian City Central Hospital of Qingdao University 230965, Taian, Shandong, China; 5 Precision and Transformation Laboratory of Traditional Chinese Medicine, The Affiliated Taian City Central Hospital of Qingdao University 230965, Taian, Shandong, China; The University of Hong Kong, Hong Kong, Hong Kong

**Keywords:** atherosclerosis, paeoniflorin, ApoE^−/−^mice, metabolomics, gut microbiota

## Abstract

Atherosclerosis (AS) is a chronic inflammatory disease and a key factor in cardiovascular disease. Paeoniflorin (PF), a monomer component extracted from red and white paeony roots, has been used for the prevention and treatment of cardiovascular diseases, but the specific mechanism remains unclear. ApoE^−/−^ mice fed a high-fat diet were randomly divided into a model group, a PF treatment group, a positive control group, and a control group fed a non-high-fat diet. The mice in the PF group were treated with 100 mg/kg (PF-100), 50 mg/kg (PF-50), and 25 mg/kg (PF-25) of PF through gavage. The positive control group was administered 10 mg/kg atorvastatin calcium tablets through gavage. After 8 weeks of gavage, PF delayed the weight gain and reduced the elevated levels of serum total cholesterol, triglycerides, and low-density lipoprotein cholesterol in ApoE^−/−^ mice, which had been induced by the high-fat diet. Concomitantly, PF alleviated aortic plaque formation and downregulated TLR4 and NF-κB expression. In terms of the underlying mechanism, PF altered linoleic acid metabolism, tryptophan metabolism, and steroid biosynthesis, as well as improved abnormalities in galactose metabolism, glycerophospholipid metabolism, and other metabolic pathways. Moreover, PF restored the dysbiosis of Firmicutes, Bacteroidota, and Actinobacteriota. Finally, it increased the abundance of *Lactobacillus*, *Bifidobacterium*, and other beneficial bacteria. Our results suggest that PF improves high-fat diet-induced AS and alleviates high-fat diet-induced intestinal barrier damage; moreover, its antiatherosclerotic effect may be related to improvements in intestinal metabolic disorders and altered abundance of microbiota.

**IMPORTANCE:**

Atherosclerosis (AS) is a chronic progressive disease mainly characterized by vascular endothelial cell damage, inflammation, and lipid deposition. Inflammatory and immune-related mechanisms play a key role in the occurrence and development of AS. Our study showed that paeoniflorin can improve blood lipid levels, reduce arterial inflammation, restore intestinal permeability, regulate metabolites, increase the abundance of beneficial flora, and alleviate the occurrence of AS. Thus, our findings may contribute to the understanding of how traditional Chinese medicine exerts its holistic effects by modulating the microbiota–metabolite axis.

## INTRODUCTION

Atherosclerosis (AS) is a chronic progressive disease that is mainly characterized by vascular endothelial cell damage, inflammation, and lipid deposition. In the process of AS development, unstable plaque rupture, vascular stenosis, platelet aggregation, and thrombosis can lead to vessel occlusion, which leads to cardiovascular diseases (CVDs), such as ischemic heart disease, stroke, and peripheral vascular disease (1). Inflammatory and immune-related mechanisms, such as the inflammation of endothelial cells and macrophages, infiltration of T cells into atherosclerotic plaques, and activation of Th17 cells, play a key role in the occurrence and development of AS (2). Currently, the first-line treatment for CVDs, including atherosclerosis, is the administration of statins (3); these agents prevent and treat AS based on their lipid-lowering ability and anti-inflammatory effects (4). However, the long-term use of statins may lead to many serious adverse effects, with rhabdomyolysis being the most serious adverse effect (5). The use of herbal medicines and their active plant components, alone or in combination with traditional Chinese medicine (TCM) therapy, is expected to reduce lipid damage (6). In vascular endothelial cells, oxidized low-density lipoprotein (OX-LDL), lysophosphatidic acid, very-low-density lipoprotein, angiotensin II, glucose, and viral infection can activate the NF-κB signaling pathway, thereby upregulating the proinflammatory factors TNF-α, IL-1, IL-8, E-selectin, and VCAM-1. The activation of the NF-κB signaling pathway can lead to vascular endothelial cell injury, thereby promoting the formation and development of AS (7).

Paeoniflorin (PF) is a monomeric monoterpenoid component that is extracted from red and white paeony roots. PF has a variety of biological activities, such as anti-inflammatory, antioxidative stress, antiplatelet aggregation, and antithrombosis effects, and is widely used in clinical practice for the prevention and treatment of cardiovascular and cerebrovascular diseases (8). PF can also enhance the autophagy of Human Umbilical Vein Endothelial Cells (HUVECs) by upregulating SIRT1, thereby reducing the apoptosis of these cells and the expression of the adhesion molecules induced by OX-LDL. Moreover, PF has a protective effect against HUVEC injury induced by OX-LDL (9). PF antagonizes the OX-LDL-induced proliferation, migration, and inflammatory action of vascular smooth muscle cells by activating HO-1; arresting the cell cycle; and inhibiting the p38, ERK1/2/MAPK, and NF-κB signaling pathways (10). In addition, PF not only ameliorates atherosclerosis by inhibiting TLR4-mediated NF-κB activation (11) but also inhibits OX-LDL-induced apoptosis and inflammation in human coronary artery endothelial cells by regulating the Wnt/β-catenin pathway (12). However, further studies are needed to determine whether PF actually acts on the NF-κB pathway to ameliorate AS in ApoE^−/−^ mice. ApoE^−/−^ mice were selected for this study due to their well-established role in modeling atherosclerosis and metabolic dysfunction. It is essential for lipid metabolism, and its deficiency leads to spontaneous hypercholesterolemia, impaired lipoprotein clearance, and atherosclerotic plaque development (13, 14).

Angiogenesis in AS plaques is a key factor for plaque instability and vulnerability. OX-LDL promotes endothelial cell angiogenesis *in vitro* and plays an important role in plaque angiogenesis (15). Research suggests that the traditional Chinese medicine *Paeonia lactiflora* may have a beneficial therapeutic effect on AS; more specifically, ligustrazine (TMP) and PF, which are the main active components of *P. lactiflora*, can alleviate atherosclerosis. After the incubation of HUVECs with OX-LDL followed by treatment with TMP, PF, or TMP and PF, the expression levels of proteins related to cell proliferation, migration, tube formation, and angiogenesis were measured. The results showed that TMP and PF attenuated OX-LDL-induced angiogenesis in HUVECs *in vitro*. Furthermore, TMP combined with PF not only inhibited the OX-LDL-induced expression of CD31, vascular endothelial growth factor (VEGF), and VEGF receptor 2 (VEGFR2) but also downregulated the OX-LDL-induced expression of Notch1, Jagged1, and Hes1. Therefore, the combination of TMP with PF can inhibit OX-LDL-induced angiogenesis in HUVECs by inhibiting the VEGF/VEGFR2 and Jagged1/Notch1 signaling pathways, which may enhance the stability of atherosclerotic plaques (16).

Researchers have examined the pathological changes and gene expression related to the reverse cholesterol transport (RCT) pathway in the aorta, liver, and gut after treating AS mice with PF. *In vitro*, RAW264.7 macrophages were used to investigate the inhibitory effect of PF on foam-cell formation and its potential to promote RCT. The results revealed that PF reduced atherosclerosis, hyperlipidemia, and hepatic steatosis. Moreover, PF may promote the occurrence of RCT by stimulating cholesterol efflux from macrophages through the liver X receptor α pathway, increasing the serum levels of high-density lipoprotein cholesterol and apolipoprotein A-I, and regulating key genes involved in RCT in the liver and intestine. In addition, treatment of ApoE mice with PF inhibited the expression of inflammation-related genes, including those encoding CD68, tumor necrosis factor-alpha, and monocyte chemoattractant protein-1, and reduced oxidative stress in the aorta and liver (17). Therefore, PF has the potential to become a more effective and safer drug for the treatment of atherosclerosis.

Metabolomics and gut microbiota have been widely used in studying cardiovascular disease and atherosclerosis (18–20). Through studying metabonomics and gut microbiota in feces, the diagnosis of diseases can be improved (21), as well as their pathogenesis and treatment methods can be better understood (22). Trimethylamine, a metabolite that is synthesized by the gut microbiota, produces the atherogenic metabolite trimethylamine-*N*-oxide. In contrast, various bile acids produced by the gut microbiota alleviate inflammation and reduce atherosclerosis (23, 24). In addition, the gut microbiota and its metabolites can regulate inflammation, immunity, cholesterol, and lipid metabolism, which are closely related to the occurrence and development of AS (25, 26). Compared with the general population, the intestinal biota of patients with AS exhibits great changes (27). Therefore, studying the relationship between the gut microbiota and its metabolites and the host can provide promising insights into the development, prognostic factors, and treatment of atherosclerosis.

## MATERIALS AND METHODS

### Experimental reagents

PF was purchased from Beijing Psaitong Biotechnology Co., Ltd (Beijing, China). Atorvastatin calcium tablets were purchased from Qilu Pharmaceutical Co., Ltd (Jinan, China). Anti-TLR4, anti-NF-κB, and anti-MyD88 primary antibodies and corresponding secondary antibodies were purchased from Abmart Shanghai Co., Ltd (Shanghai, China). High-fat feed (21% fat + 0.15% cholesterol) was purchased from Shandong Zhao Lai Biotechnology Co., Ltd (Jinan, China).

### Animals and experimental design

Animal experiments were approved by the Animal Ethics Committee of the Affiliated Taian City Central Hospital of Qingdao University (Approval No. 20240811). Six-week-old male ApoE^−/−^ mice were purchased from Beijing Vital River Laboratory Animal Technology Co., Ltd. Mice were given water and food under a controllable environment (temperature, 22℃ to ~25°C; humidity, 45% to ~55%; 12:12 h light-dark cycle). After arrival at the laboratory, the mice were fed adaptively for 1 week and then randomly divided into six groups, with eight mice in each group. One group was fed with a standard diet (control group), and the remaining five groups were fed a high-fat diet (21% fat + 0.15% cholesterol) for a total of 12 weeks. After 12 weeks, the mice in the high-fat-diet group were again randomized into five groups and administered PF at a high dose (100 mg/kg), PF at a medium dose (50 mg/kg), PF at a low dose (25 mg/kg) (17, 28, 29), atorvastatin calcium tablets (positive control drug), and normal saline (model group) through gavage. In contrast, the control group was administered the same dose of normal saline once a day for 8 weeks. At the end of the experiment, the mice were anesthetized, and echocardiography was performed to assess the blood-flow velocity and plaque formation in the heart aorta. Blood samples were collected, and serum was separated. After heart and aortic perfusion, aortic and colonic tissues, and intestinal contents were collected and stored in liquid nitrogen or tissue fixative for subsequent analysis.

### Determination of serum biochemical indicators

After the mice were anesthetized, blood was collected from the eyeball, and the upper serum was collected after centrifugation for 15 min at 3,500 rpm at 4°C. The levels of total cholesterol (TC), triglycerides (TGs), and low-density lipoprotein cholesterol (LDL-C) were measured using an automatic biochemical detector.

### Assessment of atherosclerosis

After perfusion, the aorta was collected from the mice in each group, and the adipose tissue was carefully dissected under a microscope and fixed in the tissue fixative solution for at least 24 hours. Subsequently, gross oil red O staining and hematoxylin and eosin (HE) staining were performed. For gross oil red O staining, peripheral fat was removed, and the vessels were carefully dissected longitudinally along the vessel wall using anatomical scissors. The cut blood vessels were first immersed in 60% isopropanol for 3 seconds, followed by immersion in the oil red O staining solution at 37°C in the dark for 60 min and then removed. The blood vessels were removed with forceps and immersed in 60% isopropanol to differentiate; differentiation started at 1 min until the fatty plaques in the lumen exhibited an orange or a bright-red color. Other areas remained nearly colorless, and differentiation was then terminated by washing with distilled water. The blood vessels were taken out, excess water was removed using filter paper, and the vessels were placed on a black or white background plate with a scale. For HE staining, the tissues were fixed and embedded in paraffin. The paraffin sections were deparaffinized, washed with water, and then treated with a high-definition constant staining pretreatment solution for 1 min. Next, the slices were stained with HE solutions. Finally, the slices were dehydrated and sealed, and images were acquired and analyzed.

### Western blotting was used to detect changes in inflammatory factors in atherosclerosis

After aortic dissection, the adipose tissue was carefully dissected under a microscope, and western blotting (WB) experiments were performed. The treated aorta was ground after adding lysate and protease inhibitors, and the supernatant was centrifuged and assayed for Bicinchoninic Acid Assay (BCA) content. Subsequently, 5× loading buffer was added for WB experiments aimed at detecting changes in the expression of TLR4 and NF-κB in the aorta.

### HE staining was used to observe pathological changes in colon tissue

After the mice were anesthetized, the colon was collected, intestinal contents were carefully removed, and the colon was rinsed in normal saline and then fixed in tissue fixative for at least 24 hours. Paraffin sections were deparaffinized, washed in water, and then placed into a high-definition constant staining pretreatment solution for 1 min. Subsequently, the slices were stained with HE solutions. Finally, the sections were dehydrated and mounted, and images were captured and analyzed.

### Immunohistochemistry was used to observe the expression of ZO-1, occludin, and MUC-2 in the colon tissue

Colon tissues were dehydrated in paraffin sections as described above for antigen repair and blockage of endogenous peroxidase, followed by blocking. Next, primary antibodies (anti-ZO-1, anti-occludin, and anti-MUC-2) were added and incubated with the tissues at 4°C overnight. The cells were washed three times with Phosphate Buffered Saline (PBS) for 5 min each time, followed by the addition of a secondary antibody (Horseradish Peroxidase-conjugated Secondary Antibody, HRP-conjugated) of the corresponding species and incubation for 50 min at 25°C. After washing three times with (Phosphate Buffered Saline) PBS, Diaminobenzidine (DAB) was added for color development; the positive color was brown–yellow, and the color development was stopped by rinsing with tap water. After counterstaining the nuclei with hematoxylin for 3 min, the dehydrated slides were sealed, followed by image acquisition and analysis. The nuclei stained with hematoxylin were blue, and the DAB-positive signal was brown–yellow.

### Immunofluorescence was used to observe the co-localization of ZO-1 and occludin in colon tissues

Sample pretreatment was performed as described above. After antigen repair, a hydrogen peroxide blocking histochemical pen was used to draw circles around the tissue sections, which were then placed in 3% hydrogen peroxide solution and incubated at room temperature in the dark for 25 min to block endogenous peroxidase. Then, the sections were washed three times with PBS for 5 min each time. This was followed by a 30 min serum blockage. The blocking solution was removed, the prepared primary antibody was added in a dropwise manner, and the mixture was incubated overnight at 4°C. Next, the slides were placed in PBS before washing three times for 5 min each. After the sections were slightly dried, the corresponding HRP-labeled secondary antibody was added in a dropwise manner in the circle and incubated for 50 min at room temperature. The corresponding Tyramide signal amplification (TSA) dye was added, and the tissue sections were heated in a repair box filled with antigen repair buffer in a microwave oven. Next, the second primary antibody was added and incubated overnight at 4°C; after washing, the corresponding secondary antibody was added. Finally, the nuclei were counterstained with DAPI, autofluorescence quenching was performed, and images were captured after sealing the slides.

### LC-MS-based untargeted metabolomics analysis

Solid samples (50 mg) were accurately weighed, and the metabolites were extracted using a 400 µL methanol:water (4:1, [vol/vol]) solution, with 0.02 mg/mL l-2-chlorophenylalanine as the internal standard. The mixture was allowed to settle at −10°C and treated with a high-throughput tissue crusher (Wonbio-96c; Shanghai Wanbo Biotechnology Co., LTD) at 50 Hz for 6 min, followed by ultrasound treatment at 40 kHz for 30 min at 5°C. The samples were then placed at −20°C for 30 min to allow the precipitation of proteins. After centrifugation at 13,000 × *g* at 4°C for 15 min, the supernatants were carefully transferred to sample vials for Liquid Chromatograph Mass Spectrometer (LC-MS/MS) analysis. Two microliter of sample was separated by HSS T3 column (100 mm × 2.1 mm i.d., 1.8 µm) and then entered into mass spectrometry detection. The mobile phases consisted of 0.1% formic acid in water:acetonitrile (95:5, [vol/vol]; solvent A) and 0.1% formic acid in acetonitrile:isopropanol:water (47.5:47.5:5, [vol/vol]; solvent B). The solvent gradient changed according to the following conditions: from 0 to 0.1 min, 0% B to 5% B; from 0.1 to 2 min, 5% B to 25% B; from 2 to 9 min, 25% B to 100% B; from 9 to 13 min, 100% B to 100% B; from 13 to 13.1 min, 100% B to 0% B; from 13.1 to 16 min, 0% B to 0% B for equilibrating the systems. The sample injection volume was 2 µL, and the flow rate was set to 0.4 mL/min. The column temperature was maintained at 40°C. During the period of analysis, all these samples were stored at 4°C. Variance analysis was performed on the matrix file after data preprocessing. The R package ropls tool (Version 1.6.2) was used to perform principal component analysis and orthogonal partial least squares discriminant analysis, and 7-cycle interactive validation was used to evaluate the stability of the model.

### Gut microbiota

The colonic contents were rapidly stored in liquid nitrogen after collection and then stored at −80°C. Absolute quantification using 16S rRNA amplicon sequencing was performed by Majorbio Bio-Pharm Technology Co., Ltd. (Shanghai, China). Total microbial genomic DNA was extracted using the E.Z.N.A. Soil DNA Kit (Omega Bio-tek, Norcross, GA, U.S.) according to the manufacturer’s instructions. The quality and concentration of DNA were determined using 1.0% agarose gel electrophoresis and a NanoDrop2000 spectrophotometer (Thermo Scientific, United States). Twelve different spike-in sequences with four different concentrations (10^3^, 10^4^, 10^5^, and 10^6^ copies of internal standards) were added to the sample DNA pools. The spike-in sequences consisted of conserved regions that were identical to those of selected natural 16S rRNA genes, and the artificial variable regions shared nucleotide sequences with negligible identity with the public databases, which functioned as an internal standard and facilitated absolute quantification across samples.

The hypervariable V3–V4 region of the bacterial 16S rRNA gene was amplified using the primer pair 338F (ACTCCTACGGGAGGCAGCAG) and 806R (GGACTACHVGGGTWTCTAAT) on a T100 Thermal Cycler PCR instrument (BIO-RAD, USA). The PCR mixture included 4 µL of 5 × Fast Pfu buffer, 2 µL of 2.5 mM deoxy-ribonucleoside triphosphate (dNTPs), 0.8 µL of each primer (5 µM), 0.4 µL of Fast Pfu polymerase, 10 ng of template DNA, and ddH_2_O to a final volume of 20 µL. The cycling conditions used for PCR amplification were as follows: initial denaturation at 95°C for 3 min; followed by 27 cycles of denaturation at 95°C for 30 s, annealing at 55°C for 30 s, and extension at 72°C for 45 s; and a single final extension at 72°C for 10 min. The samples were then maintained at 10°C until halted by the user. The PCR product was extracted from the 2% agarose gel, purified using the PCR Clean-Up Kit (YuHua, Shanghai, China) according to the manufacturer’s instructions, and then quantified using Qubit 4.0 (Thermo Fisher Scientific, USA).

The optimized sequences were denoised using the DADA2 (https://qiime2.org) plugin in the Qiime2 (https://qiime2.org) pipeline with recommended parameters, which obtains single-nucleotide resolution based on error profiles within samples. DADA2 denoised sequences are usually called amplicon sequence variants (ASVs). Then ASVs assigned to spike-in sequences were filtered out, and reads were counted. A standard curve (based on read counts versus spike-in DNA copy number) for each sample was generated, and the quantitative abundance of each ASV in a sample was determined.

### Data analyses

The experimental data above were analyzed and plotted using GraphPad Prism 9.5.1 software. To compare multiple groups, one-way Analysis of Variance (ANOVA) was used. The results are expressed as mean ± SEM. A significance threshold of *P* < 0.05 indicated a statistically significant difference among the groups.

## RESULTS

### PF improved the excessive blood-flow velocity in the heart arteries of atherosclerotic mice

**Fig 1:**
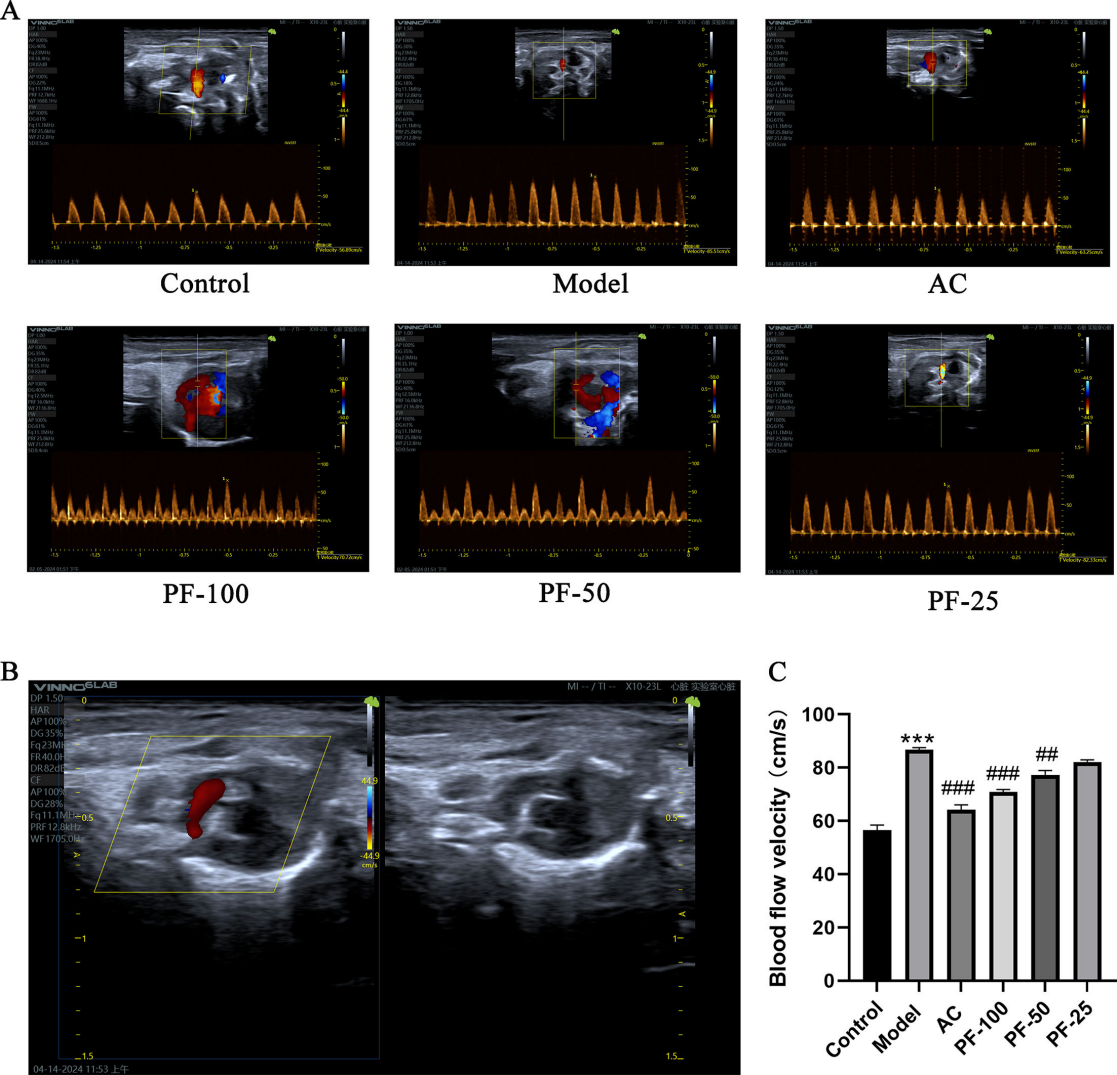
Echocardiograms of mice. (**A**) Representative echocardiograms of mice in each group. (**B**) Representative map of plaque in the mice in the model group. (**C**) Statistical analysis of blood-flow velocity of mice in each group. * *P* < 0.05, **P < 0.01, and ***P < 0.001, model vs control; ^#^ *P* < 0.05, ^##^* P* < 0.01, ^###^* P* < 0.001, and^ ####^* P* < 0.0001 (AC, PF-100, PF-50, and PF-25) vs model.

As depicted in Fig. 1, the vascular wall of the control mice was smooth, the lumen was well filled with blood flow, and the blood-flow velocity was relatively slow. In the model group, the vessel wall was rough, hypoechoic, and hyperechoic, and mixed echoic plaques of different sizes were observed on the vessel wall; moreover, the lumen was not well filled with blood flow, and the blood-flow velocity was significantly increased. Compared with the model group, the AC and PF-100 groups exhibited less stenosis, less plaque, and a lower blood-flow velocity.

### PF reduced the weight gain and dyslipidemia induced by the high-fat diet in mice

**Fig 2:**
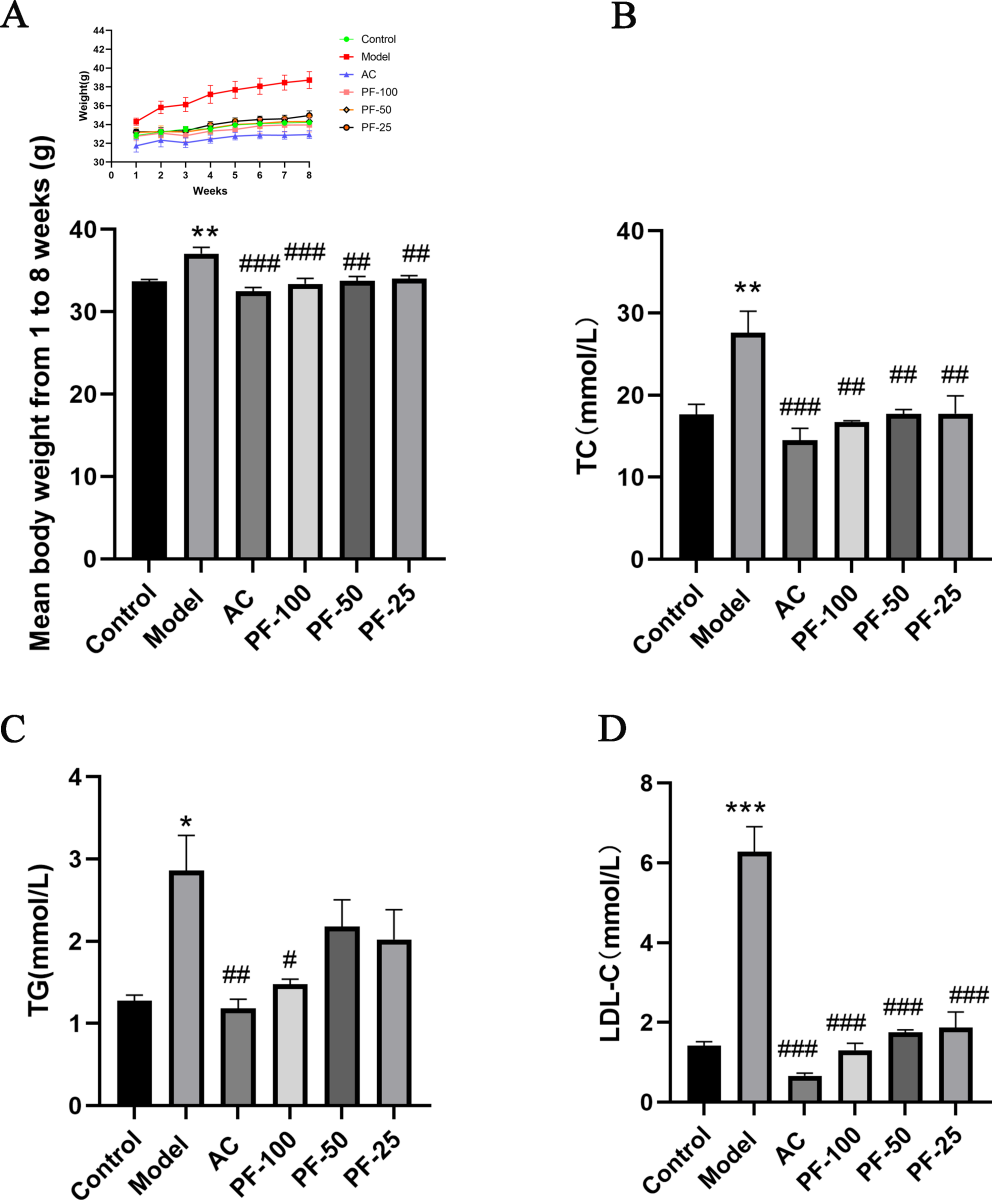
Changes in body weight and serum markers in mice. (**A**) Mean changes in the body weight of mice at 8 weeks (*n* = 6). (**B–D**) Serum levels of markers in the mice after 8 weeks of treatment (*n* = 3). Data are expressed as mean ± SEM. *P < 0.05, **P < 0.01, and ***P < 0.001, model vs control; ^#^ *P* < 0.05, ^##^ *P* < 0.01, and ^###^ *P* < 0.001 (AC, PF-100, PF-50, and PF-25) vs model.

After the onset of the intragastric administration of PF, the changes in the body weight of the mice in each group were recorded weekly, as shown in Fig. 2A. After 8 weeks, compared with the control group, the body weight of the mice in the model group was significantly increased, whereas in the PF-100, PF-50, PF-25, and AC groups, the high-fat diet induced body weight gain was significantly reduced.

Lipid levels were measured in the mice after 8 weeks of treatment, as shown in Fig. 2B through D. Compared with the control group, the high-fat diet resulted in significant increases in serum TC, TG, and LDL-C levels. AC and PF at high, medium, and low doses significantly reduced the levels of TC and LDL-C, whereas the TG content was reduced only in the AC and high-dose PF groups.

### PF improved the degree of atherosclerotic lesions induced by a high-fat diet in mice

**Fig 3:**
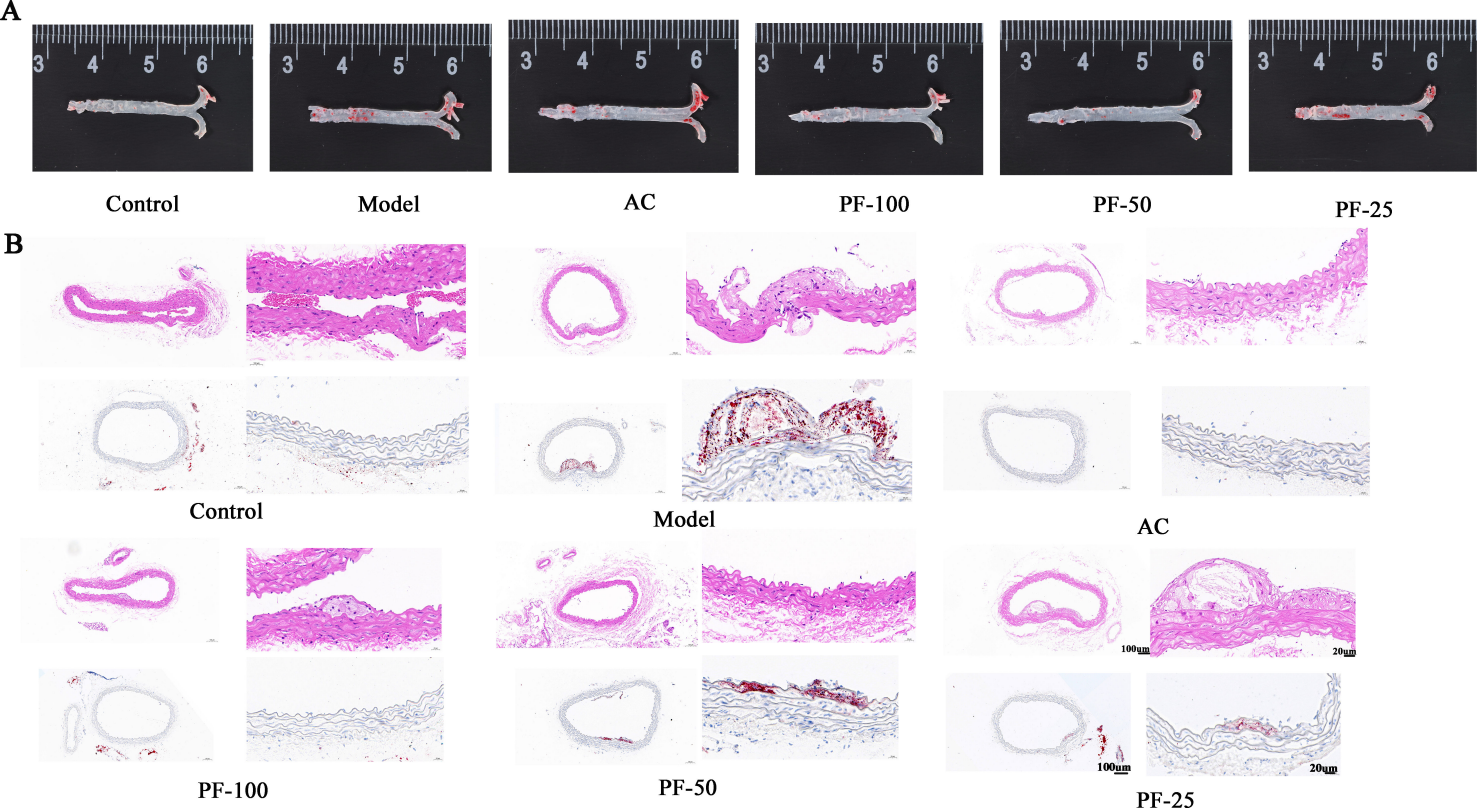
Effect of PF on the development of atherosclerotic lesions in ApoE^−/−^ mice. (**A**) Parts of the mice in each group were grossly stained with oil red. (**B**) Representative images of cross-sections (10× and 40×) of atherosclerotic plaques, as detected using HE staining. Representative images of cross-sections (10× and 40×) of atherosclerotic lipid deposition, as detected using oil red staining.

To assess the degree of atherosclerotic lesions in mice, their arteries were stained with HE and oil red stain, as shown in Fig. 3. HE staining revealed that compared with the control group, the local structure of the vessel wall in the model group was unclear, the internal elastic plate and the internal elastic plate in the media were broken, the endothelial cells were missing in many areas, the smooth muscle cells in the media were arranged irregularly, and a greater number of cells were swollen. However, there were no obvious pathological changes in the high, medium, and low PF and AC groups. Oil red staining revealed that the intima of the model group was slightly thickened. Moreover, plaque formation and a greater amount of lipid deposition were observed in a small area of the vascular intima. Lipids were stained in red, which confirmed the success of AS induction. No obvious plaque formation was observed and lipid deposition in the aorta of mice in the AC, PF-100, PF-50, and PF-25 groups, indicating improvement in atherosclerotic lesions.

### PF downregulated the expression of inflammatory markers in atherosclerosis induced by a high-fat diet in mice

**Fig 4:**
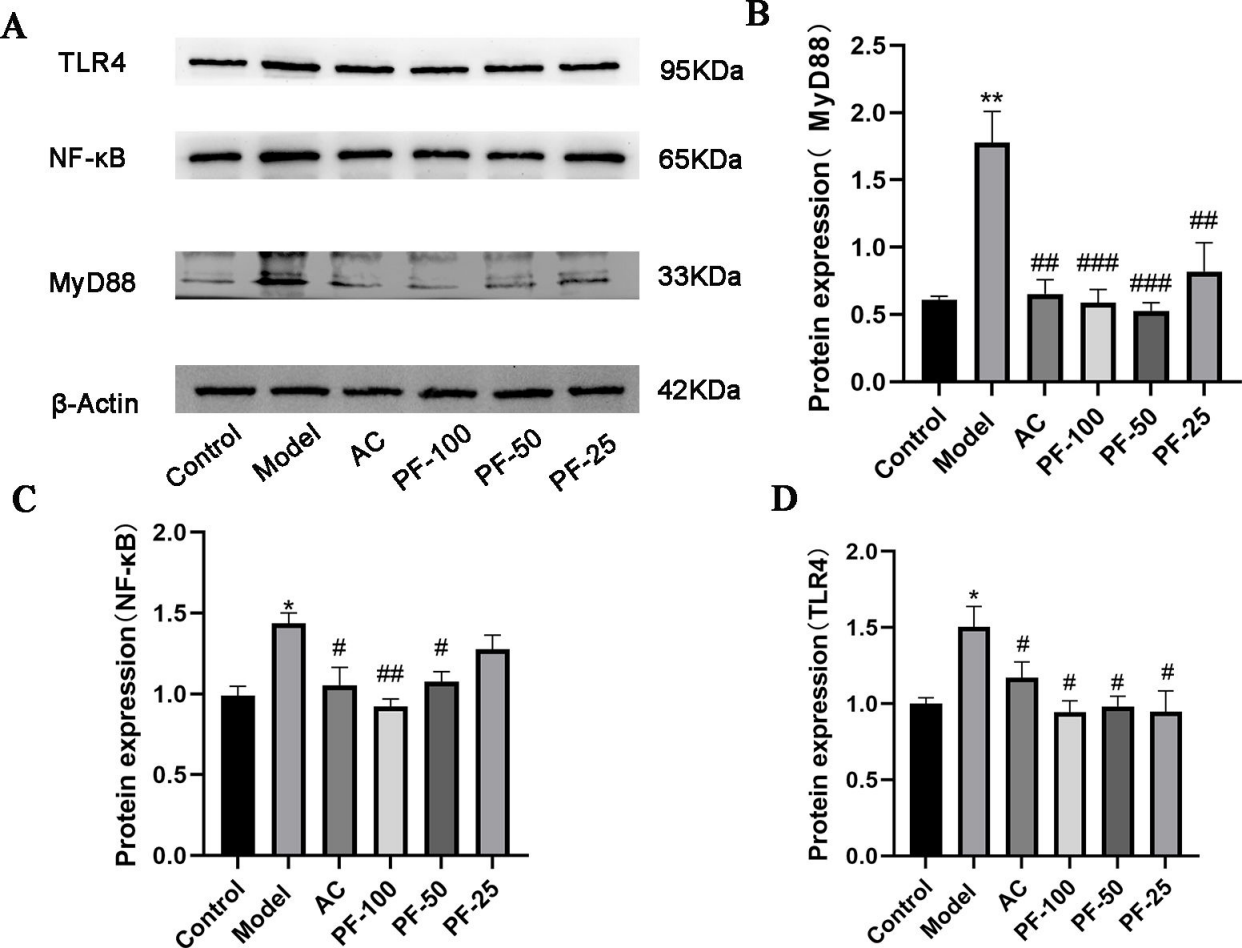
Expression of proteins in the NF-κB pathway in mouse arteries. (**A**) WB image. (**B–D**) Expression of the MyD88, NF-κB, and TLR4 proteins. * *P* < 0.05, **P < 0.01, and ***P < 0.001, model vs control; ^#^* P* < 0.05, ^##^* P* < 0.01, and ^###^* P* < 0.001 (AC, PF-100, PF-50, and PF-25) vs model.

Considering the important role of inflammation in AS, the expression of key proteins involved in the NF-κB pathway was examined in arteries. As depicted in Fig. 4, compared with the control group, the expression of NF-κB, TLR4, and MyD88 was increased significantly in the arteries of the model group after the administration of the high-fat diet. Moreover, a significant inhibitory effect was observed after treatment with PF, with the PF-100 dose yielding the best results.

### PF restored the intestinal injury induced by the high-fat diet and reversed the downregulation of the intestinal tight junction proteins ZO-1, occludin, and MUC2 caused by the high-fat diet

**Fig 5:**
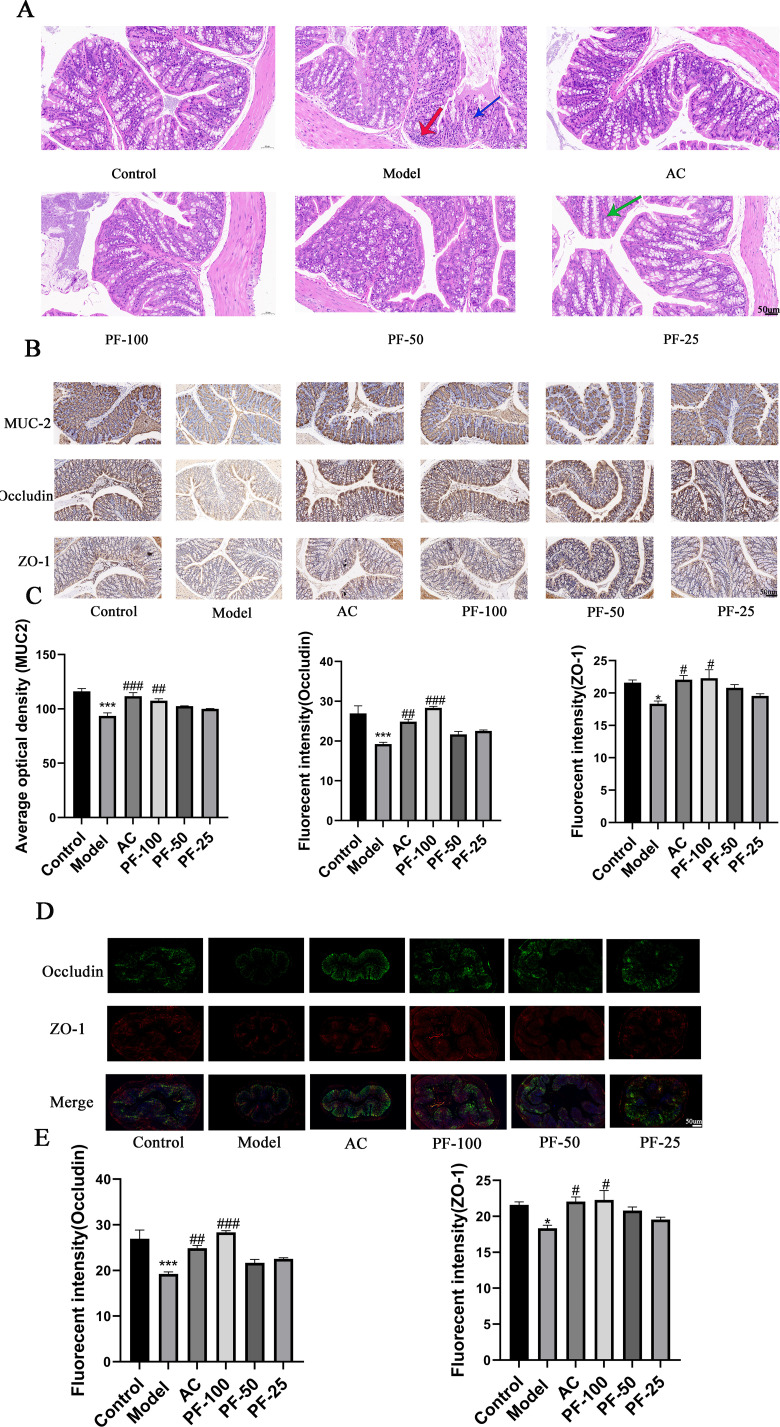
Effects of PF on the intestinal lesions and permeability induced by a high-fat diet. (**A**) Representative cross-section (40×) of mouse colon tissue showing pathological changes, as detected through HE staining. In the model group, a small amount of nuclear pyknosis in mucosal epithelial cells (blue arrow) and occasional focal aggregation of lymphocytes in the lamina propria (red arrow) was observed. In the PF-25 group, a small number of blood vessels were congested and dilated in the lamina propria (green arrow). (**B and C**) Statistical map of intestinal immunohistochemical expression and average optical density expression in the mice in each group. (**D and E**) Statistical diagram of intestinal immunofluorescence expression and fluorescence intensity expression in mice in each group. * *P* < 0.05, **P < 0.01, and ***P < 0.001, model vs control; ^#^* P* < 0.05, ^##^* P* < 0.01, and ^###^* P* < 0.001 (AC, PF-100, PF-50, and PF-25) vs model.

HE staining was used to observe the pathological injury of the cecum of mice in each group. The results showed that in the control group, the mucosal layer of the intestinal tissue protruded into the intestinal lumen to form folds, and the folds were abundant. In the model group, the cytoplasm of upper mucosal cells exhibited eosinophilia, a large number of intestinal glands in the lamina propria were straight or round tubes that were densely arranged, and the number of goblet cells was reduced compared with the normal group. Moreover, compared with the model group, the AC and PF groups showed varying degrees of improvement in these pathological injuries. The mice in the AC, PF-100, and PF-50 groups showed improvements in the infiltration of inflammatory cells, and the PF-25 group displayed minor blood vessel congestion and dilatation in the lamina propria (Fig. 5A).

ZO-1, occludin, and MUC2 are key tight junction proteins in epithelial cells; they are secreted proteins that are produced by goblet cells and play an important role in the maintenance of the function of epithelial and endothelial barriers. The quantitative analysis of these three proteins in the colon of mice using immunohistochemistry showed that, compared with the control group, the three proteins were significantly downregulated in the colon of mice in the model group; furthermore, after AC and PF administration, they were significantly increased. The content of ZO-1 in the PF-100 and PF-50 groups was increased. However, the PF-100 group alone showed an increase in the levels of occludin and MUC2 (Fig. 5B and C). To further examine the effects of the high-fat diet and PF on the intestinal tract of mice, ZO-1 and occludin were selected for immunofluorescence staining (Fig. 5D and E). Compared with the control group, these proteins were significantly downregulated in the colon of mice in the model group, and their content was significantly increased in the PF-100 and AC groups. These results showed that the effect observed in the PF-100 group was better than that in the PF-50 and PF-25 groups, which provided a basis for subsequent metabolomics experiments.

### PF modulated fecal metabolites in ApoE^−/−^ mice

#### Principal component analysis and orthogonal partial least squares discriminant analysis

The results showed that the model group could be significantly separated from the remaining groups, as it had significant changes in endogenous metabolites compared with the other groups. Concomitantly, the Quality Control (QC) sample aggregation was obvious, indicating that the instrumental method was stable. Each scatter point represented a sample, and the color and shape of the scatter points represented different groups. A closer distribution of sample points indicated more similar types and contents of metabolites in the samples. Conversely, a more distant sample indicated a greater difference in its overall metabolic level. Subsequently, model vs control and metabolites between AC, PF-100, PF-50, and PF-25 vs model were analyzed for model reliability; the Q2 values of pairwise comparison groups were all greater than 0.5, indicating that the model had good explanatory and predictive ability. Next, a permutation test was performed to illustrate the reliability of the test, with the number of tests set to 200. The slopes of R2 and Q2 in pairwise comparison groups were greater than 0, and the intercept was less than 0.05, indicating that the model was robust and reliable and did not overfit, and the data were reliable (Fig. S1).

#### Differential metabolite and metabolic pathway analysis

**Fig 6:**
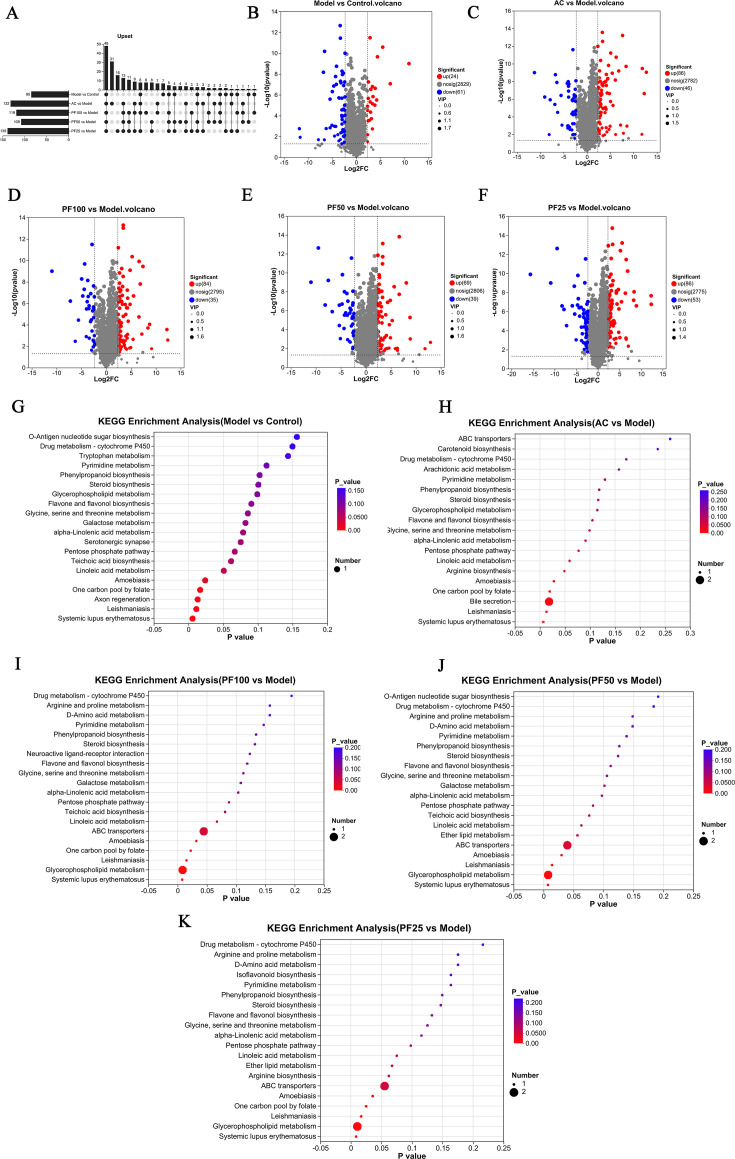
Map of differential metabolites and metabolic pathways. (**A**) Upset diagram of differential metabolites in each group. (The horizontal bar graph on the left shows the number of substances with contrast differences between the two groups, and the circles show the substances common to these metabolic sets, corresponding to the number of bars on the top). (**B–F**) Model vs control analysis and volcano diagram of differential metabolites in the AC, PF-100, PF-50, and PF-25 vs model analysis. (**G–K**) Model vs control and AC, PF-100, PF-50, and PF-25 vs model metabolic pathway bubble plots.

After the analysis described above, combined with the results of the statistical analysis of unit variables and multivariate variables, 205 differential metabolites with a VIP value of >1, *P* < 0.05, and a fold change up/down of 5 times were screened out. Compared with the control group, 85 metabolites were altered in the model group; of these 85 metabolites, 61 were downregulated, and 24 were upregulated. Compared with the model group, 119 metabolites were altered in the PF-100 group, among which 84 were upregulated and 35 were downregulated. A total of 108 metabolites were altered in the PF-50 group, among which 69 were upregulated and 39 were downregulated. A total of 139 metabolites were altered in the PF-25 group, among which 86 were upregulated and 53 were downregulated. Finally, a total of 132 metabolites were altered in the AC group, among which 86 were upregulated and 46 were downregulated. The results of differential metabolites were visualized in the form of a volcano diagram and an Upset diagram, as shown in Fig. 6A through F.

Differences in metabolites in annotated metabolic pathways were assessed using KEGG database analysis (https://www.kegg.jp/kegg/pathway.html). The Python software package SciPy (v1.0.0) was used for pathway enrichment analysis, and Fisher’s exact test was used to obtain the most relevant biological pathways to explore the metabolic mechanism of action of PF in the treatment of atherosclerosis in the intestine. The main metabolic pathways were screened based on the following screening conditions: *P* value < 0.05 and a high impact value score, as shown in Fig. 6. The analysis showed that in the model group, the high-fat diet interfered with linoleic acid metabolism. Compared with the model group, the main metabolic pathways in the PF-100, PF-50, and PF-25 groups were linoleic acid metabolism and ether lipid metabolism. The main metabolic pathways in the AC group included linoleic acid metabolism and arginine biosynthesis. The analysis described above indicates the likelihood that PF improves the occurrence of atherosclerosis through linoleic acid metabolism and ether lipid metabolism (Fig. 6G through K).

### PF remodeled the gut microbiota in ApoE^−/−^ mice

A total of 644,353,800 bases were obtained in the 338F_806R region, and a total of 2,147,846 single-end reads were obtained. The DADA2 plugin in the QIIME2 (https://qiime2.org) workflow was used to reduce the noise of the sequencing reads. The steps of noise reduction included filtering noise and correcting sequence errors, removing chimeras and single sequences, and sequence duplication to obtain high-resolution ASVs for subsequent analysis. A total of 682,560 sequences were generated from 18 samples after DADA2 (https://qiime2.org) denoising, and 6,823 ASVs were obtained.

#### Alpha diversity and beta diversity

**Fig 7:**
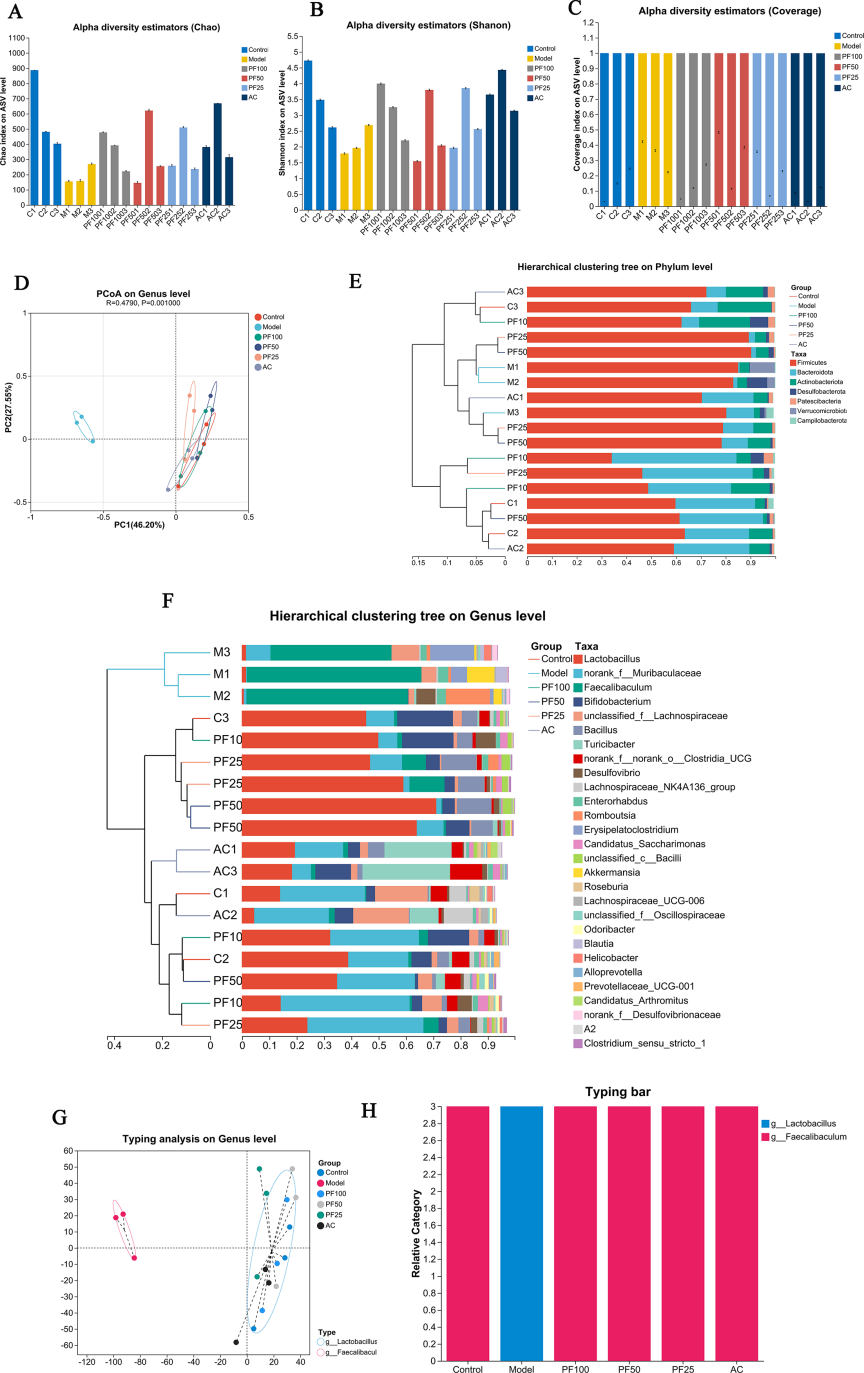
Alpha and beta diversities of gut microbiota. (**A–C**) Chao, Shannon, and coverage indices. (**D**) principal co-ordinates analysis （PCoA） score at the genus level. (**E and F**) Phylum and genus level, species composition, and abundance in each group. (**G and H**) The distribution of microbiota in each group was analyzed at the genus level.

To further examine community richness and diversity in these mice, the species richness index Chao, the diversity index Shannon, and the coverage index were analyzed in all samples under the alpha diversity index. Compared with the control group, the Chao and Shannon indices of the model group were significantly decreased, indicating that the richness and diversity of intestinal microorganisms in ApoE^−/−^ mice were significantly reduced after high-fat-diet intake. The coverage value indicated that the coverage of sample species in each group was good (Fig. 7A through C).

Under beta diversity, PCoA (Bray–Curtis, ANOSIM) was used to examine the differences in bacterial community composition. The PCoA spots in the same group clustered well, indicating that the bacterial community composition was similar in the same group of samples. At the genus level, samples from the model group were far from samples from other groups, whereas samples within the group were close, indicating significant differences between the groups. The control, PF-100, PF-50, PF-25, and AC groups were separated by distance, indicating that the bacterial community structure was different among different populations (Fig. 7D).

Cluster analysis and dendrograms were used to analyze the species in each group. At the phylum level, the species composition of Firmicutes, Bacteroidota, Actinobacteriota, Desulfobacterota, Patescibacteria, Verrucomicrobiota, and Campylobacterota was higher in each group. At the genus level, the species composition of Lactobacillus, Muribaculaceae, Faecalibaculum, Bifidobacterium, Lachnospiraceae, Bacillus, Turicibacter, and Clostridia_UCG-014 was abundant (Fig. 7E and F).

At the genus level, the intestinal types of each group were mainly concentrated in g__Lactobacillus and g__Faecalibaculum. Among them, the model group was concentrated in g__Faecalibaculum, whereas the control, PF, and AC groups were mainly concentrated in g__Lactobacillus. These results suggest that PF can improve atherosclerosis; however, it can alter the distribution of intestinal-type bacteria to a certain extent. The genus of the biota after drug administration tended to be similar to that of the control group (Fig. 7G and H).

#### Community composition analysis

**Fig 8:**
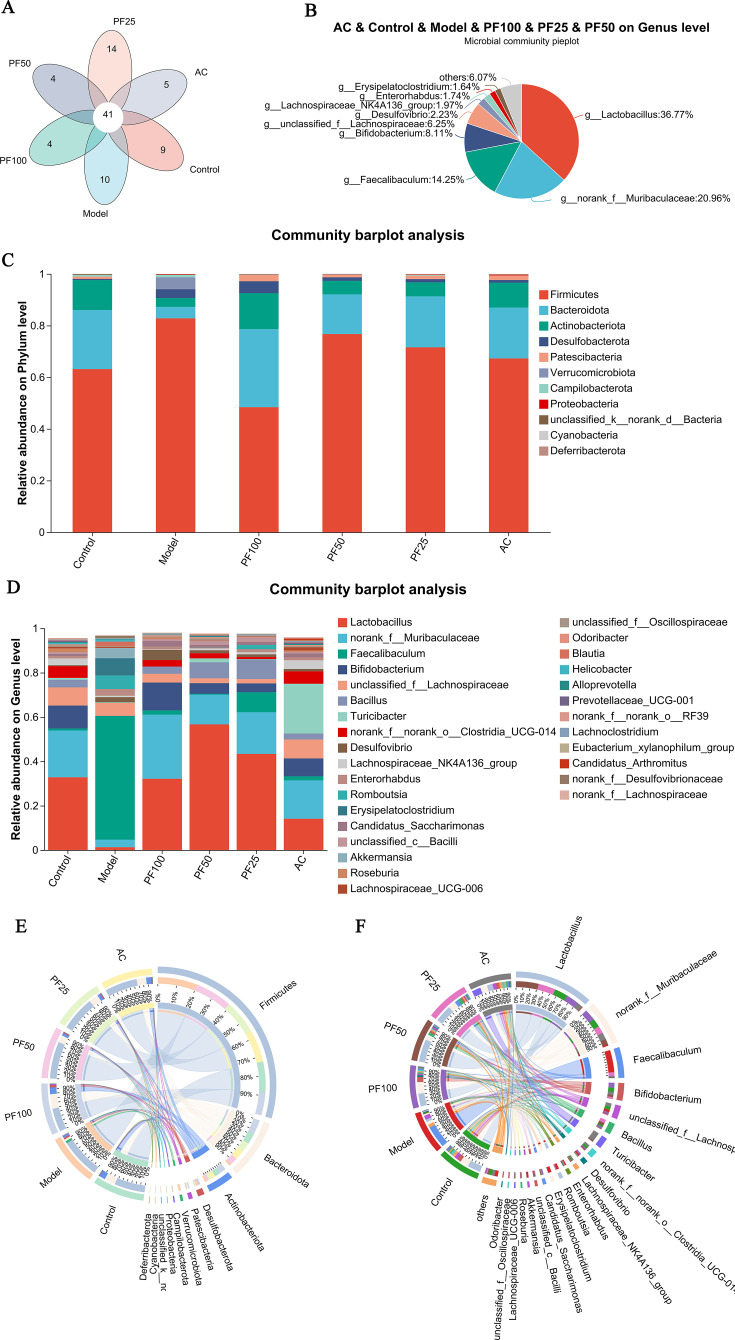
Map of the community composition analysis. (**A and B**) Venn diagram and pie chart of community composition. (**C and D**) Bar plot of the community at the phylum and genus levels. (**E and F**) Circos at the phylum and genus levels.

The number of unique and shared microorganisms among the multiple groups was counted at the genus level via annotation analysis. Among them, the number of species shared by all groups was 41 (Fig. 8A and B). At the phylum level, Firmicutes, Bacteroidota, and Actinobacteriota were the dominant bacteria in each group, and the proportion of these three phyla was greater than 90% in each group. Among them, the abundance of Firmicutes in the model group was significantly increased and showed a downward trend after drug administration in each group, especially in the PF-100 group. The abundance of Firmicutes in the AC group was close to that in the control group. The abundance of Bacteroidota was significantly decreased in the model group and increased in each group after drug administration, especially in the PF-100 group. At the genus level, Lactobacillus, f__Muribaculaceae, Faecalibaculum, Bifidobacterium, and f__Lachnospiraceae were the top five genera. Compared with the control group, the number of Lactobacillus in the model group was significantly decreased, the number of Lactobacillus in the PF-50 group was most significantly increased, and the proportion of Lactobacillus in the PF-100 group was closest to that in the control group. The abundance of Faecalibaculum was significantly increased in the model group and significantly decreased after drug administration in each group. Similarly, f__Muribaculaceae and Bifidobacterium were significantly decreased in the model group and increased to varying degrees after drug administration in each group (Fig. 8C and D).

At the phylum level, Firmicutes, Bacteroidota, and Actinobacteriota were the most abundant species in each group. Firmicutes was mainly found in the model group (20%). Bacteroidota was mainly present in the control group (20%) and PF-100 group (27%). Actinobacteriota was mainly present in the control group (24%), PF-100 group (28%), and AC group (20%).

At the genus level, the biota species mainly consisted of Lactobacillus, f__Muribaculaceae, Faecalibaculum, Bifidobacterium, and f__Lachnospiraceae. Lactobacillus was mainly concentrated in the PF-50 group (31%) and PF-25 group (24%), whereas f__Muribaculaceae was mainly concentrated in the control group (21%) and PF-100 group (28%). Faecalibaculum was mainly found in the model group (80%). Bifidobacterium was mainly detected in the control group (26%) and PF-100 group (32%), with no expression in the model group (0%). f__Lachnospiraceae was mainly concentrated in the control group (27%) and AC group (28%).

In conclusion, Firmicutes and Faecalibaculum abundances were closely associated with high-fat-diet-induced AS models. The changes in the abundance of Bacteroidota, Bifidobacterium, and f__Muribaculaceae may be closely related to the anti-AS effect of PF. The changes in the abundance of Actinobacteriota may be closely related to the anti-AS effects of PF and atorvastatin (Fig. 8E and F).

#### Species-difference analysis

**Fig 9:**
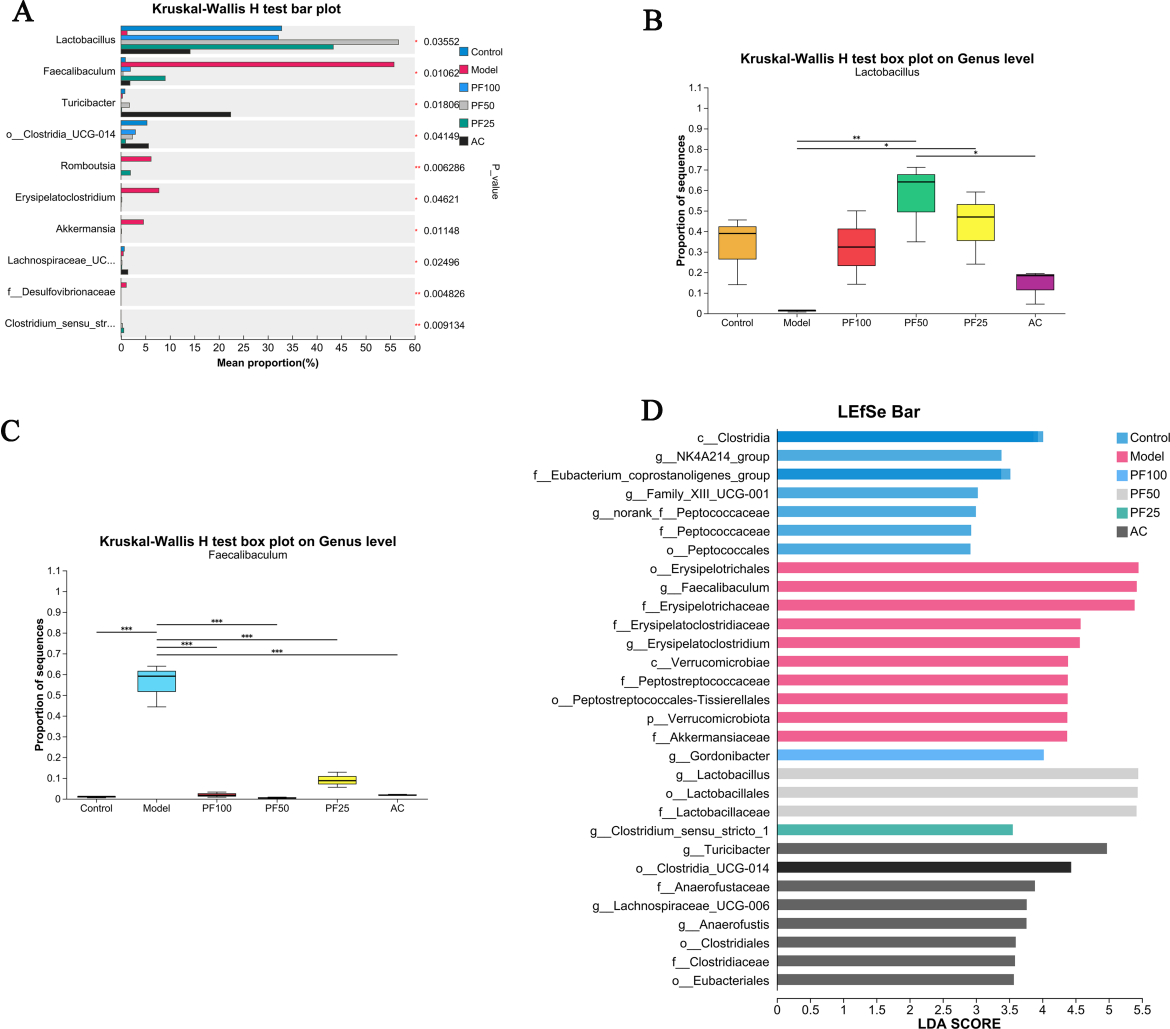
Analysis of species diversity. (**A**) Differences in the average relative abundance of the same species among different groups. (**B and C**) Statistical analysis of *Lactobacillus* and *Faecalibaculum* at the genus level. (**D**) Linear discriminant analysis Effect Size （LEfSe）analysis at the phylum to genus level. * *P* < 0.05, **P < 0.01, and ***P < 0.001.

At the genus level, *Lactobacillus* and *Faecalibaculum* were significantly different among the multiple groups of samples. As depicted in Fig. 9, for *Lactobacillus*, a significant difference was observed between the model group and the PF-50 group (*P* < 0.01). For *Faecalibaculum*, significant differences were observed between the control group and model group (*P* < 0.001) as well as between the PF treatment and AC groups and model group (*P* < 0.001).

An LEfSe analysis was performed to further identify biomarkers with an abundance that was significantly different between groups at the phylum to genus levels. In total, 10 significant taxa were enriched in the control group: c__Clostridia, g__NK4A214_group, and f__Eubacterium_coprostanoligenes_group. In contrast, 23 significant taxa were enriched in the model group: O__Erysipelotrichales, g__Faecalibaculum, f__Erysipelotrichaceae, g__Erysipelatoclostridium, and f__Erysipelatoclostridiaceae (Fig. 9).

#### PICRUSt2 function prediction and Spearman’s correlation analysis

**Fig 10:**
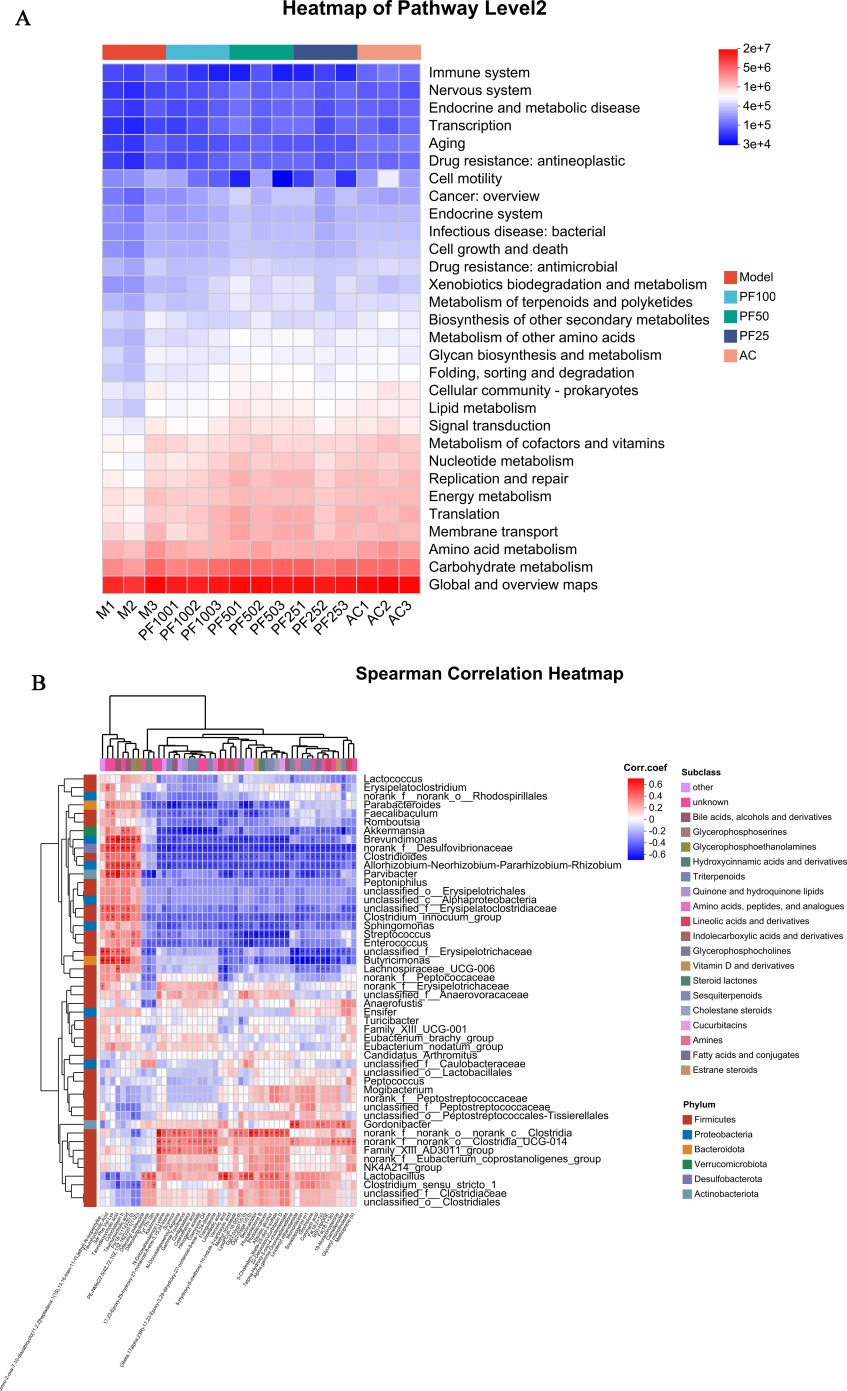
Prediction analysis of intestinal metabolites and intestinal microorganisms. (A) PICRUSt2 functional prediction (the distribution of KEGG functional abundance in different samples is displayed using heat maps, which intuitively show the distribution of the main dominant functions in different samples). Note: The abscissa is the sample name (or group name), and the ordinate is the MetaCyc pathway function name. The color gradient of the color block is used to indicate the changes in the abundance of different functions in the sample/group, and the figure is the value represented by the color gradient. (B) Spearman’s correlation analysis heat map with microbial classification (right) and metabolites (bottom). A cluster dendrogram based on the correlation coefficient is presented on the left and top. The color bar represents the magnitude of the correlation coefficient: the closer the absolute value is to 1, the higher the correlation of metabolites, with red indicating a positive correlation and blue indicating a negative correlation; darker color indicates a stronger correlation, and the asterisk indicates a significant correlation between metabolites and microorganisms: *P < 0.05, **P < 0.01, and ***P < 0.001.

PICRUSt2 (https://github.com/picrust/picrust2/) predicted the functional profile of the microbial communities at level 2 of the KEGG pathway. As shown in Fig. 10, the immune system, nervous system, transcription, endocrine and metabolic disease, and endocrine pathways were involved in the atherosclerosis induced by a high-fat diet in mice. Genes involved in the biosynthesis of other secondary metabolites, metabolism of other amino acids, cell growth and death, lipid metabolism, carbohydrate metabolism, amino acid metabolism, and other genes were closely related (Fig. 10A).

To explore the relationship between different gut microbiota and different metabolites in AS development, Spearman’s correlation analysis was performed (Fig. 10B). The development of AS was associated with taurodeoxycholic acid, vernolic acid, kashmirine, momordicinin, camellenodiol, Parvibacter, Brevundimonas, Butyricimonas, Lactobacillus, and Akkermansia.

## DISCUSSION

In the present study, we investigated the effects of PF on high-fat diet-induced AS in ApoE^−/−^ mice as well as the underlying mechanisms. Changes in serum lipids, arterial inflammatory factors, intestinal biota composition and structure, fecal metabolites, and KEGG enrichment pathways were observed after PF administration. In addition, the correlation between gut metabolism and AS was determined. PF administration not only improved dyslipidemia and arterial inflammation but also increased the diversity and richness of the gut microbiota. In addition, PF treatment modulated specific gut microbiota, fecal metabolites, and metabolic pathways associated with CVD. The differences in fecal metabolites between groups were also strongly associated with differences in gut microbiota and atherosclerotic damage.

In recent years, AS has been reported as an important risk factor for CVDs. The study has reported that the lack of *ApoE* expression combined with a high-fat diet can lead to an increase in triglyceride levels and an imbalance in cholesterol homeostasis, thereby promoting the formation of aortic and carotid artery branch root/main branch AS plaques and inducing cardiovascular and cerebrovascular diseases (30). An elevated plasma LDL-C concentration is a major risk factor for atherosclerotic cardiovascular disease. Lowering blood lipids contributes to the occurrence and development of AS (31).

Furthermore, PF administration significantly reduced atherosclerotic plaques and lipid deposition, suggesting that PF has a similar anti-atherosclerotic effect to AC. Previous studies have found that NF-κb pathway-related proteins are closely associated with the occurrence of atherosclerosis (32, 33). Therefore, we examined the expression of the TLR4/MyD88/NF-κb proteins in the arteries of mice, and the results showed that the expression levels of these three proteins were significantly increased in the model group. After PF administration, the expression of the TLR4/MyD88/NF-κb proteins was significantly inhibited. PF may inhibit the inflammatory response via the TLR4/MyD88/NF-κB pathway to attenuate the occurrence of atherosclerosis; therefore, the specific underlying mechanism warrants further study.

ZO-1, MUC2, and occludin are barrier-related proteins, among which ZO-1 is essential for mucosal repair (34). However, MUC2 deficiency can lead to colonic inflammation, indicating that Muc2 is essential for colonic protection (35). Similarly, occludin is closely related to the permeability of the mouse colon (36). Therefore, in this study, we used immunohistochemistry and immunofluorescence to observe the changes in intestinal permeability in mice. Our results indicated that the expression of these three proteins was significantly decreased in the colon of the mice in the model group, whereas the expression of these three proteins was significantly increased after the administration of PF and AC, suggesting that a high-fat diet may cause changes in colonic permeability. However, PF administration reversed the occurrence of such conditions.

Metabolomics has been routinely used for the discovery of biomarkers and for the study of system-level effects of metabolites and subtle changes in biological pathways, providing methods for understanding the underlying mechanisms of various diseases (24, 37). Pyruvate dehydrogenase kinase has been found to regulate vascular inflammation in atherosclerosis and increase cardiovascular risk (38). In this study, we collected mouse feces for metabolite studies. The results showed that the main metabolic pathways of PF in the treatment of atherosclerosis were linoleic acid metabolism, steroid biosynthesis, and galactose metabolism. This provides a basis for our subsequent intervention on the metabolism of atherosclerosis.

In recent years, the gut and its accompanying microbial communities have attracted attention due to their important role in physiological and pathological events in the host (39). Changes in the gut microbiota are associated with many disease states, including CVDs (40). Reduced diversity and richness of gut microbial species have been reported to increase the risk of AS; our study aligns with previous findings showing that HFD reduced the diversity of gut microbiota (41). However, PF treatment significantly increased the diversity and richness of intestinal microbial species in ApoE^−/−^ mice. Furthermore, changes in the bacterial community composition of different specific microbial species at different taxonomic levels were also strongly associated with CVD. Firmicutes and Bacteroidetes are the two dominant phyla in the human gut microbiota. An increase in Firmicutes is positively associated with CVDs and increased risk of obesity and AS, whereas Bacteroidetes has the opposite effect (42). The PCoA results of the present study indicated that the gut microbiota composition of the model group differed significantly from that of the remaining groups. At the phylum level, the abundance of Firmicutes was significantly increased in the model group. PF and AC treatment not only reduced the abundance of Firmicutes but also increased the abundance of Bacteroidetes, with the PF-100 group exhibiting a more significant result.

In addition, both *Lactobacillus* and *Bifidobacterium* have been widely reported to be beneficial to human health by effectively regulating oxidative stress and lipid metabolism and improving the occurrence of AS (43, 44). In the present study, *Lactobacillus* was highly present in the PF-50 and PF-25 groups, whereas *Bifidobacterium* was mainly concentrated in the PF-100 group, suggesting that PF administration can increase the abundance of *Lactobacillus* and *Bifidobacterium*. In addition to the composition of the gut microbiota, increased attention has been paid to the overall function and metabolism of microbial communities. The PICRUSt2 function prediction and Spearman’s correlation analysis suggested that the occurrence of atherosclerosis in mice is closely related to the immune system, nervous system, transcription, other functional genes, and taurodeoxycholic acid. The promising effects of PF on metabolic pathways and gut microbiota highlight its potential as a novel therapeutic agent for AS. Further research should explore the precise mechanisms by which PF regulates linoleic acid and tryptophan metabolism, potentially identifying new molecular targets for AS intervention. In addition, investigating the synergistic effects of PF with probiotics or prebiotics to enhance Short-chain fatty acids (SCFA)-producing bacteria (e.g., *Lactobacillus* and *Bifidobacterium*) can optimize gut microbiota modulation and anti-inflammatory efficacy. Ultimately, PF may emerge as a multitargeted, microbiota-modulating agent in the treatment of atherosclerosis and related metabolic disorders. While our sample size limited the detection of rare taxa, the observed trends in dominant microbial patterns remain informative. These preliminary findings warrant validation in larger-scale studies to fully characterize the microbial diversity.

### Conclusion

Our study showed that PF can improve blood lipid levels, reduce arterial inflammation, restore intestinal permeability, regulate metabolites, increase the abundance of beneficial biota, and alleviate the occurrence of AS. Moreover, our findings contribute to the understanding of how TCM exerts its holistic effects by modulating the microbiota–metabolite axis. Overall, these results suggest that PF is beneficial for cardiovascular health and has favorable efficacy in the treatment of AS. In the future, we will continue to explore the mechanism through which PF plays an important role in CVDs.

## SUPPLEMENTAL MATERIAL

Figure S1 msystems.00990-25-s0001.pdf PCA and OPLS-DA.

ASM does not own the copyrights to Supplemental Material that may be linked to, or accessed through, an article. The authors have granted ASM a non-exclusive, world-wide license to publish the Supplemental Material files. Please contact the corresponding author directly for reuse.
