# Supplementary material for: Paeoniflorin improves atherosclerosis by regulating the gut microbiota and fecal metabolites
Source: mSystems. 2025 Aug 15;10(9):e00990-25. doi: 10.1128/msystems.00990-25 (PMC12455941; doi:10.1128/msystems.00990-25)
Supplement: Figure S1 — PCA and OPLS-DA. [file msystems.00990-25-s0001.pdf]

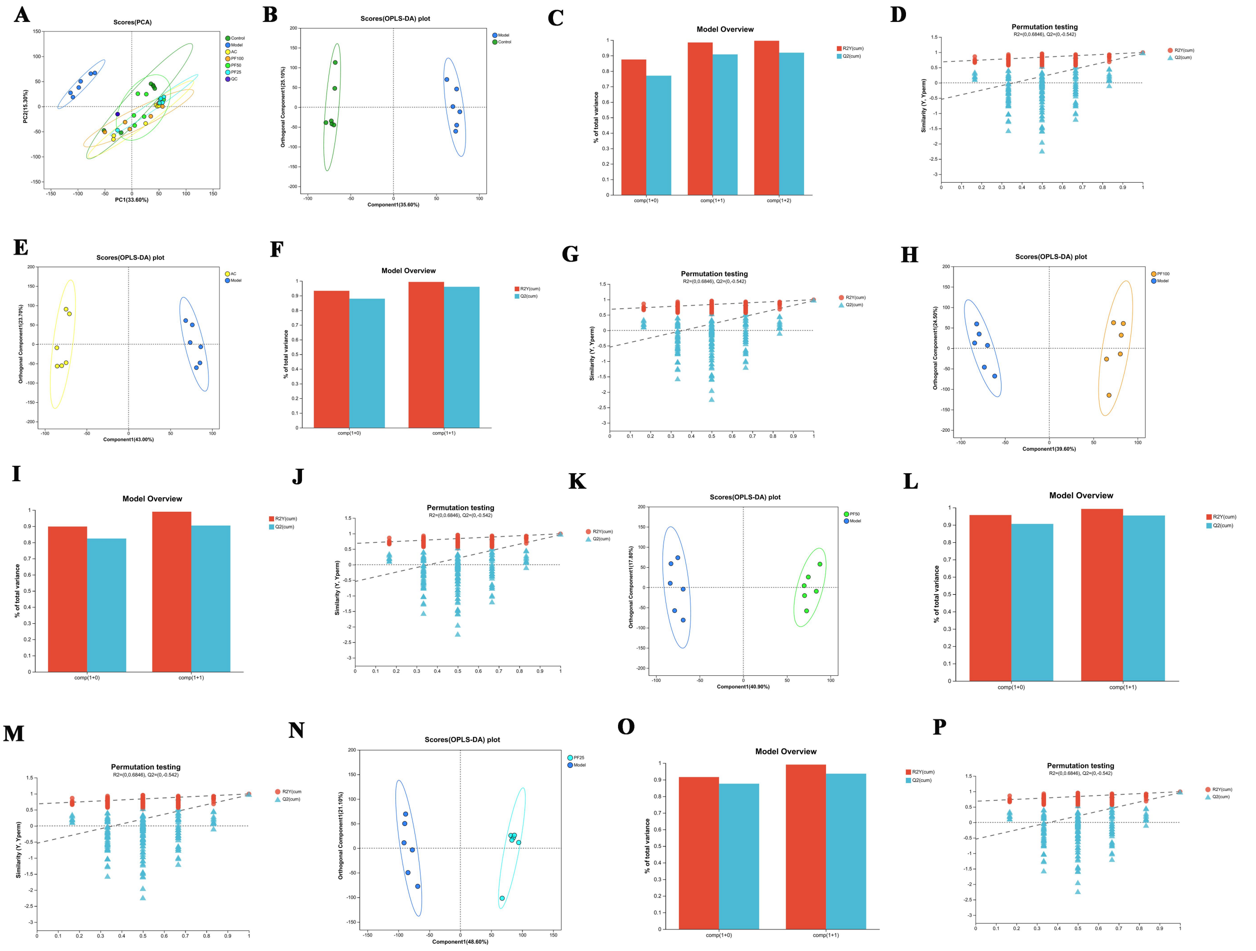

Figure S1 Principal component analysis and orthogonal partial least squares discriminant analysis. (A) PCA score plots of Control, Model, AC, PF-100, PF-50 and PF-25 groups. (B-D) OPLS-DA score plot, PLS-DA permutation test plot, PLS-DA principal component number selection plot of Model VS Control group. (E-G) AC VS Model group OPLS-DA score plot, PLS-DA permutation test plot, PLS-DA principal component number selection plot. (H-J) PF-100 VS Model group OPLS-DA score plot, PLS-DA permutation test plot, PLS-DA principal component number selection plot. (K-M) PF-50 VS Model group OPLS-DA score plot, PLS-DA permutation test plot, PLS-DA principal component number selection plot. (N-P) PF-25 VS Model group OPLS-DA score plot, PLS-DA permutation test plot, PLS-DA principal component number selection plot.
